# Acknowledgment to the Reviewers of *Biology* in 2022

**DOI:** 10.3390/biology12010137

**Published:** 2023-01-16

**Authors:** Biology Editorial Office

**Affiliations:** MDPI AG, St. Alban-Anlage 66, 4052 Basel, Switzerland

High-quality academic publishing is built on rigorous peer review. *Biology* was able to uphold its high standards for published papers due to the outstanding efforts of our reviewers. Thanks to the efforts of our reviewers in 2022, the median time to first decision was 17 days and the median time to publication was 40 days. Regardless of whether the articles they examined were ultimately published, the editors would like to express their appreciation and thank the following reviewers for the time and dedication that they have shown *Biology*:
A. K. M. Mominul IslamLaura GrassiAaron B. MortonLaura KaravirtaAarti BainsLaura Merras-SalmioAbbas MalandishLaura RighettiAbdallah IbrahimLaura SannaAbdallah Tageldein MansourLaura Sterian Terian WardAbdellatif ChaoutiLaura Teresa Hernández-SalazarAbdel-Majid KhatibLauren WeidnerAbderraouf Ben AbderrahmanLaurence DinanAbdollah AllahverdiLaurent DuchateletAbdul Awal Chowdhury MasudLaurent PenetAbdul Rashid AzizLawrence SowersAbdul Rehman NiaziLéa CabrolAbdullah ShaitoLeah GheberAbdullah TariqueLeah M. Panek-ShirleyAbdullahi AlhassanLeah M. PyterAbe HiroyaLeah R. ReznikovAbel PiquerasLeandro ÁlvarezAbhayraj S. JoshiLeandro Rodríguez-VieraAbhik SahaLeandro SantiagoAbhimanyu ThakurLech KotwickiAbhinav AeronLee FrelichAbhinav JainLee-Kuen ChuaAbhinav SurLeena StraussAbhinava MishraLei HuangAbid Ali KhanLen GoodmanAbigail Ngugi-DawitLenka KlčováAbigail R. BlandLeo J. CumminsAbolfazl NarmaniLeon GrayferAbraham Escobedo-MoratillaLéon ReubsaetAbraham Wall MedranoLeonardo AvillaAbu HazafaLeonardo BrunettiAchinta SannigrahiLeonardo José Gil BarcellosAda Margarida Correia Nunes Da RochaLeonardo L. FrutteroAdam KreutzerLeonardo MessiasAdam ŁukowskiLeonardo RestaAdam McDonnellLeonel PereiraAdam PyzikLeonidas IoannouAdam SiemieńskiLeslie BuckAdam W. PotterLetizia BernardoAdarsh K. GuptaLevente CzeglédiAdeel MalikLi ChenAdemola Emmanuel AdetunjiLiana PlesAdi PerelmanLiana RomaniukAdijailton Jose De SouzaLiang MaAdil Othman AbdullahLiang ZhangAditi PrabhakarLiang ZhongAdmilton Gonçalves De OliveiraLifat RahiAdnan MustafaLifei WangAdrian Canizalez-RomanLi-Hsin WuAdrian IndreicaLihua LiuAdriana F. SestrasLikang ChinAdriana GrandisLi-Kwan ChangAdriána LiptákováLila KariAdriana MorarLili QuAdriana RojasLilian SosaAdrianna MostowskaLiliana KiczakAdrianne BendichLiliana V. BelokopytovaAdriano MollicaLiliane PetriniAgata GrytaLilla TuriákAgata KlejmanLily J. D. DeMarsAgata MikolajczykLimin WuAgata Olszewska-WiddratLin WalkerÁgnes CsivincsikLinda GailīteAgnieszka A. KaczorLinda HouhamdiAgnieszka BanasLinda PetersAgnieszka BossowskaLing HeAgnieszka ChwałczyńskaLing LiAgnieszka Ewa WiącekLingling JinAgnieszka KrawczenkoLinlin GuoAgnieszka KuźniarLinn HoffmannAgnieszka PartykaLinnet Roque-RomeroAgnieszka RichertLiqiang LuoAgnieszka Synowiec-WojtarowiczLiqing ZangAgnieszka WolińskaLiqiong GuoAhan DalalLira GaysinaAhmad AfifyLisa GorskiAhmad MirzaLisa M. RileyAhmed BadrLise Román MoltzauAhmed ElaswadLixin LvAhmed MaghrabyLluvia De Abril Alexandra Soriano-MelgarAhmed Mohamed Ali KhalilLong GuoAhmed Mohamed SaadLong ZhengAhmed MostafaLonny R. LevinAhmed SalmanLora Petrie-HansonAhmet TopalLoredana NicolettiAida BiancoLoren FardellAilyn FadriquelaLorena Di PietroAimilia MantzouLorena Garcia Del RioAindrila MukhopadhyayLorenz S. NeuwirthAjay KumarLorenza VantaggiatoAjay PalLorenzo FaggioniAjaz AhmadLorenzo MariAjinkya KawaleLorenzo PutzuAkath SinghLoretto Contreras-PorciaAkira HashimotoLori A. FischerAkira KitamuraLorraine ArcherAkira MatsumuraLouise M. SoanesAkira TakamataLourdes Magadalena Orejuela-EscobarAklank JainLu DongAkoad Mohamed ElhassanLu WangAkram Hosseinian SerajehlooLuan De Souza LeiteAkriti SrivastavaLuca AmbrosinoAlain PerretLuca IncrocciAlain RamanantsoanirinaLuca LombardoAlan GiraldoLuca PetrignaAlan M. YoungLuca PoliAlba Camacho-CardeñosaLuca SalvatiAlba RoccoLuca TeseiAlbert ZinkLucas DelmonicoAlberto Arias-PérezLucia Beltran-CamachoAlberto ChighineLucia CastelliAlberto CollaretaLucia NastiAlberto EspíLuciano BossoAlberto Férriz-ValeroLucyna KirczukAlberto GallegosLudmila RudiAlberto García GómezLudwig WagnerAlberto Jorge Oliveira LopesLuigi CipolloniAlberto Rubén OsellaLuigi De MasiAlberto SalomoneLuigi EspositoAlberto TroccoliLuigi FortunaAldo NicosiaLuis Augusto Eijy NagaiAlegría-Lertxundi IkerLuís C. N. Da SilvaAlejandro Martínez-RodriguezLuis GandíaAlejandro Miguel Figueroa-LópezLuís M. FélixAlejandro Schcolnik-CabreraLuís MargalhoAleksander StułaLuis Miguel Garcia-SeguraAleksandr PoliakovLuís Valença PintoAleksandra Elzbieta Badaczewska-DawidLuisa BergaminAleksandra GolobLuisa GarofaloAleksandra JarońLuiz Felipe Lima Da SilveiraAleksandra KrólikowskaLuiz Marcos Garcia GonçalvesAleksandra Piechota-PolanczykLujiang QuAleksandra S. KristoLukas BrauschAleksandra ŻebrowskaLukas HartlAleksei A. PochekutovLukas PfeiferAleksei KaledaŁukasz A. PoniatowskiAleksey ZaitsevŁukasz KaczmarekAlen SoldoLukasz KajtochAlena SalákováŁukasz MigdałAleš PavlíkLuke Mangaliso DuncanAlessandra BergadanoLunda ShenAlessandra MontecuccoLusheng FanAlessandra SaporitiLutendo F. MugwediAlessandra StacchiottiLuu Thai DanhAlessandra VaralliLuyen VuAlessandro CrocoliLydia TsoutsoubiAlessandro LeparuloLyudmila KamburskaAlessandro MariottiMaaike KockxAlessandro MarroniMacarena Gomez-LiraAlessandro MengarelliMaciej JaneczekAlessandro ProvenzaniMaciej JedlińskiAlessandro RigaMaciej MaciejczykAlessandro ScalaMaciej SkorackiAlessio AlesciMaciej W. SochaAlessio FacchinMackenzie MazurAlessio MartinoMaddalena CurciAlessio MetereMadelaine RomainAlessio VenezianoMadelaine S. H. GiercAlex Al KhouryMadjda BellamriAlex Buoite StellaMadjid SoltaniAlex MichelsMagali ReyAlexander BetekhtinMagdalena HagovskaAlexander DimitrovMagdalena KaraśAlexander FaizulinMagdalena KowalczykAlexander G. DvoretskyMagdalena KrbotAlexander I. AlexandrovMagdaléna KrulováAlexander JaisMagdalena LisAlexander KuprinMagdalena MaselloAlexander LangMagdalena MichelAlexander OtahalMagdalena PogorzelecAlexander RuchinMagdalena SenzeAlexandr Alexandrovich KolobovMagdalena SzeligaAlexandra Díez-MéndezMagdalena Tudrujek-ZdunekAlexandra KinnbyMagdalena Weber-RajekAlexandra Peregrina LavínMagdolna SzantoAlexandra RibaritsMahar FatimaAlexandra TeixeiraMahdi HosseinzadehAlexandra TsidulkoMahesh NeupaneAlexandre BoyerMahmoud YaishAlexandre BuffetMahrous Abdelbasset IbrahimAlexandre RibeiroMaiana Silva ChavesAlexandros LaiosMakoto EndoAlexandros MakisMakoto HasegawaAlexandru Nicolae UngureanuMalcolm PageAlexei SlepzofMalene Friis HansenAlexey K. PiskunovMalgorzata A. WitekAlexey KolodkinMałgorzata AdamczukAlexey MaximovMałgorzata Buksińska-LisikAlexey PakhnevichMałgorzata Chmielewska-KrzesińskaAlexey SuminMałgorzata GrzesiukAlexios Vlamis-GardikasMałgorzata Korczak-AbshireAlexis GalanisMałgorzata Kotula-BalakAlexis JanosikMalgorzata KucinskaAlexis StamatikosMałgorzata Lasik-KurdyśAlfonsas VainorasMałgorzata LenartowiczAlfonsina MilitoMallikharjuna KoriviAlfonso Aguilar-PereraMaman TurjamanAlfonso NavasMan ZhouAlfredo AttisanoManas Kumar BagAlfredo GueraManasés González-CortazarAlfredo MartínezMancy TongAlfredo Téllez ValenciaManendra Babu LankadasariAli BahadurManfred M. VietenAli BoolaniManfredi RizzoAli MarieManickam AshokkumarAli ShokriManish TripathiAli ZarrabiManoj MajeeAlia TelliMansoor HussainAlice BongManuel Chavarrias OlmedoAlice Elena GheneaManuel J. Lis AriasAlice FerrariManuela MontanaroAlicia E. ToranzoManuela PlutinoAlicia María VignattiMara P. SteinkampAlicia Perera-CastroMarc DeanAlicia Sánchez-MendozaMarc FolcherAlicja KowalczykMarc LochbaumAlin CiobicaMarcel KunrathAlina KuryłowiczMarcel Matei DudaAlisha RenfroMarcello Di MartinoAlistair V. NunnMarcello TagliaviaAllah DittaMarcelo CoertjensAllan G. RasmussonMarcelo Tigre MouraAllison BarryMárcia AraújoAlma Netzahuatl-MuñozMarcin DębowskiAlmut KöhlerMarcin JakubowskiAlok Ranjan SahuMarcin KucińskiAlon KahanaMarcin ŁyszkiewiczÁlvaro Briz-RedónMarcin WnukAlvaro Hermida-AmeijeirasMarcio Martinello SanchesÁlvaro Santana-GarridoMarco Alfonso PerroneAlvaro YogiMarco ArculeoAlvin LeeMarco CamperaAmanda Marchi MaioranoMarco MalagutiAmar ParvateMarco SalvatoriAmar SinghMarco ScortichiniAmaranta FocardiMarco Van BelkumAmarish YadavMarcos Edel Martinez-MonteroAmer AhmedMarcos RozasAmerigo GiudiceMarcos ZeddaAmir Madany MamloukMarcus RobinsonAmira WanasMardik LeopoldAmirah GozaliMarek GancarzAmirhamed BakhtiarydavijaniMarek LecewiczAmirmasoud AhmadiMarek LubośnyAmit JaiswalMarek NahajowskiAmit KumarMarek PieszkaAmna SaharMarek SkrzypskiAmnart PoapolathepMarek WesołowskiAmr A. MohamedMargaret JonesAmrita SalviMargarida DuarteAmutha RamadasMargarita BakaevaAmy K. WrayMargarita Díaz MartínezAmy StrydomMargarita Gozalo DelgadoAna Alexandra GoraMargherita CorrentiAna AlexandreMargherita NeriAna Carolina LuchiariMaria Amélia Martins-LouçãoAna Cecilia AnzulovichMaria AnastasiouAna Jurinjak TusekMaria Anele RomeoAna Laura De La GarzaMaria Antonietta PanaroAna Luísa Coderniz PicançoMaria BartolomeuAna Luiza De Carvalho FelippiniMaria Carmen ValorosoAna Paula TagliariMaria Caterina TurcoAna PomboMaria Cristina BudaniAna RodrigoMaria Cristina CaroleoAna Sanchez-RodriguezMaria Da Luz MathiasAna Sofia Pereira PedroMaria Daniela SilvaAna Sofia Ruivo AlvesMaria De Jesús Rovirosa-HernandezAna Teresa Corte-RealMaría Del Mar Sánchez-FuentesAna ValadoMaría Del Mar YlleraAna Virginia FilgueirasMaría Dominguez CastellanoAnandharajan RathinasabapathyMaría E. CasadoAnandrao Ashok PatilMaría Elizbeth Alvarez-SanchezAnastasia G. MitseaMaria Erminia GenoveseAnastasia HyrinaMaria Fernanda AchinellyAnastasia N. SergeevaMaria Filomena CaeiroAnastasia StefanakiMaria FrosiniAnastasya AnashkinaMaria GarciaAnca BobircaMaria GiordanoAnca Corina FarcasMaria Giovanna BelcastroAnca NicolauMaria GreabuAnca Oana DoceaMaría GuiradoAnca PanaitescuMaria Helena SilvaAnca-Roxana PetroviciMaria IgottiAndré FialaMaria Isabel Ribeiro DiasAndré Lopes FulyMaria IskraAndré RamalhoMaria Jesus Vinolo GilAndre RodackiMaria João SousaAndré V. RubioMaría José Bautista-LlamasAndrea BalliniMaría José Castro-AlijaAndrea BellodiMaria KuzminaAndrea BonicelliMaria L. MateusAndrea ButeraMaria Lorena AbateAndrea CeciMaria Luisa CalabròAndrea DincherMaria Maddalena Del Gallo Di RoccagiovineAndrea FabbriMaría Maricela Carrasco-YépezAndrea GualaMaría Mendoza-MuñozAndrea L. DiCarloMaría MorazaAndrea PoloMaria Moreno CatalaAndrea S. SequeiraMaría Pérez-MarcosAndrea SabatiniMaria PratAndrea SambriMaria Rosaria De FalcoAndrea ScozzafavaMaria Rosaria De MiglioAndrea TarozziMaria Rosaria RicciardiAndrea VanniniMaría Rúa-AlonsoAndreas DoetschMaria S. MuntyanAndreas HerbstMaría Sofía UrbietaAndreas KruegerMaria Soto-GironAndreas ManiosMaria SpletterAndreas WestphalMaria SztachelskaAndreea CosoveanuMaría Teresa Cubo SánchezAndreia De CarvalhoMaria Teresa RebeloAndres E. CarrilloMaria Vesna NikolicAndrés FloresMaria VlioraAndrêssa Silva FernandesMaria-Dolores GarciaAndrew CookeMariamena ArbitrioAndrew FryMarian BrzozowskiAndrew H. JheonMarian ProorocuAndrew HooymanMariana Buranelo EgeaAndrew J. KolarikMariana Pereira AntoniassiAndrew LaneMarianna Flora TomaselloAndrew LavenderMarianna KulkaAndrew N. MillerMariano González-PérezAndrew OrkneyMarie SjögrenAndrew OrtagliaMarie-Christine JonesAndrew YoungMariella BarattiAndrey Alexandrovich LegalovMarija MeznaricAndrey CherstvyMarika MassaroAndrey ElchaninovMarika PellegriniAndrey ErstMarina DanalacheAndrey FrolovMarina KhodanovichAndrey I. AzovskyMarina M. S. Cabral-PintoAndrey PlotnikovMarina P. IsaevaAndrey SinjushinMarina PaolucciAndriy NovikovMarina PennaAndrzej HutorowiczMarina RobasAndy DavisMarina Stamenković-RadakAnella SaggeseMarina VilenicaAneta OlszewskaMario Bernardo-FilhoÁngel Canal-AlonsoMario MauroÁngel ChimeneaMario Mendoza-SagaonAngel J. MatillaMário ToméAngel Miramontes CarballadaMario ValentinoAngela BaldanzaMarion JohnsonAngela BoggeroMarisa Gabriela RepettoAngela Di SommaMarisa P. SárriaAngela M. Garcia SanchezMarius MasiulisAngela MusellaMariusz SzmytAngela RoggeroMariza VorsterAngelina Lo GiudiceMark BurnsAngelo ArmandiMark Elisabeth Theodorus WillemsAngelo BañaresMark PageAngelo CiacciulliMark PatchettAngelo Del MondoMark PittelkowAngelo GazzanoMark SutherlandAngelo LavanoMark TupperAngelo SabagMark W. LuckenbachAniello FrancescoMarko KumrićAniello MaieseMarko R. CincovićAnil Kumar YadavMarkus SchlegelAnil VermaMarleen KlannAnindya GangulyMarlon Moscoso-MartínezAnita GiglioMarta BełkaAnita ZachariasMarta GrabowskaAnithadevi Kenday SivaramMarta OpalińskaAnja MeyerMarta RusekAnjan Kumar PradhanMarta SarkozyAnket SharmaMarta Seco-CerveraAnkita ChatterjeeMarta SzachniukAnn H. RossMarta Torres-TorrillasAnna Adamiok-OstrowskaMartha Gabriela Gaxiola-CortesAnna Bilska-WilkoszMartha Martínez-GordilloAnna Boroń-KaczmarskaMartha ValiadiAnna CalarcoMartin A. MasuelliAnna CariboniMartin BenavidesAnna CedroMartin BergerAnna Cleta CroceMartin C. ArosteguiAnna DittrichMartin HellerAnna GałązkaMartin KonvickaAnna Jakubska-BusseMartin L. DuennwaldAnna Jezierska-DomaradzkaMartin MüllerAnna Karin RosbergMartin ŠlachtaAnna KitaevaMartin TeraaAnna MajewskaMartina BerettaAnna MichnikMartina BuonannoAnna NowinskaMartina CoppariAnna Ploch-JankowskaMartina Crispo BenedettoAnna Szczerba-TurekMartina F. MarongiuAnna WorthmannMartina HerrmannAnna ZwierzchowskaMartina LeopizziAnnalisa CappellaMartina MuraroAnnamaria CiminiMartine MeunierAnnamária ErdeiMartiño Cabana OteroAnnamaria LocascioMartino PaniAnne J. AndersonMartti VastamäkiAnne KonkleMartyna Ewa Maszota-ZieleniakAnne LaurençonMartyna TrzeszczAnne Prigent-TessierMarwa ElgendyAnnie PerezMaryam Borhani-HaghighiAnte IvankovićMaryam KeshavarziAnte RađaMaryam TofangchihaAnthony HuangMaryline C. BossusAnthony J. BarleyMaryline Magnin-RobertAnthony M. GeorgeMaryna SteynAnthony MaxwellMaryse DeleheddeAnthony RoweMarzena Mogielnicka-BrzozowskaAnthony ValverdeMarzia Di DonatoAntje BanningMarzia VasarriAntoine H. ChaanineMarzieh SalimiAntoine TouzéMasafumi KameyaAnton KrivopalovMasahiko TerajimaAnton O. SvininMasahito NakanoAnton SobinovMasahito YamagataAntonella ArgentieroMasami YoshimuraAntonella CostaMasaya AkashiAntonella GuzzonMasayuki SatakeAntonella OliveroMasoumeh Kourosh AramiAntonia Dolores Asencio MartinezMassimo CristofaroAntonieta RuizMassimo GuarnaAntonino GermanaMassimo SalviAntonino MignanoMassimo TusconiAntonino SpanuMasumi SuzuiAntonio Alberto Rodríguez SousaMáté VirághAntonio AversaMatej TomasAntonio BarbatoMatej VajdaAntonio BelcariMathieu DenoelAntonio BellastellaMathieu E. A. PoulicekAntonio CicchellaMathieu MahillonAntonio E. Aquino JuniorMatías MorínAntonio G. Checa GonzálezMats C. RaneboAntonio GiovinoMatteo Bruno LodiAntonio León-VazMatteo MartinaAntonio MinopoliMatteo RipaAntónio NogueiraMatteo RipamontiAntonio PalazzoMatthew DaughertyAntonio PalladinoMatthew DonaduAntonio Pérez-PérezMatthew J. JorgensenAntonio SantagostiniMatthew KlukowskiAntonio SermekMatthew MahAntonio Simone LaganàMatthew S. LehnertAntonio TiberiniMatthias BallauffAntonio ZuorroMatthias EckhardtAnudishi TyagiMatthias ElgetiAnurag SunpapaoMatthias KropfAnwesha KhanraMatthias ScheweAnyu BaoMatthias SipiczkiApostolos P. ApostolidisMattia DominoniArati SridharanMattias MandorferAravinthkumar JayabalanMatúš ČomaArbaz SajjadMatúš HrivnákArcesio Salamanca CarreñoMaulin PatelArchana TiwariMauricio Javier LozanoAre Hugo PrippMaurizio FalconiArend Jan BothMaurizio ForteArgiris S. SymeonidisMaurizio G. AbrignaniAri IglesiasMauro AngelettiArian Correa-DíazMauro ChiviteArianne MessermanMauro CorradoArieh EdenMauro FasolaAriel KorenMauro FoisArif SiddiquiMauro GiuffrèArindam GhatakMauro LombardoAristotelis C. PapageorgiouMauro MarzoratiArjan VissinkMauvis A. GoreArjun Prasad TiwariMawieh A. HamadArkadiusz ArtyszakMaxime BillotArkadiusz UrbańskiMaximilian SchmachtArkaitz Castañeda BabarroMayukh GuhaArmando Vega-LoṕezMayumi NishiArmen MulkidjanianMazhar HussainArnaud MartinMazhar IqbalArnulfo Hernan Nava-ZavalaMd. Kamal HossainArpan KusariMd. Moshfekus Saleh-E-InArpita KulkarniMegan RombergArtem M. ProkofievMehmet Hamit OzyaziciogluArtem NedoluzhkoMehmet Ilyas CosacakArthur De Sá FerreiraMehmet SarierArthur ShapiroMehrnaz ShoushtarianArthur Z. GüthMei HongArtur BanachMei-Jin GuoArtur MayerhoferMeijing WangArtur YakimovichMeisheng YiArturo CalzadaMelanie DupuisAsef RedwanMelanie KalischukAsha ThomasMelanie L. GrahamAshenafi F. BeyiMelissa D. JordanAshfaqur RehmanMemoona RamzanAshish KothariMenderes KabadayıAshley B. C. GoodeMeng WuAshok KumarMeng ZhuAshutosh BahugunaMengxing LiAshutosh PandeyMennattAllah Hassan AttiaAshutosh TripathiMercè Fernández MiróAshwini Khanderao JadhavMian HuangAsif AliMichael Bording-JorgensenAsma MaqboolMichael Douglas KallerAsma OsivandMichael F. HynesAssane Hamidou AbdoulayeMichael FineAssunta BertacciniMichael GareljaAstrid DeryckereMichael GollobAtashi SharmaMichael GreenAtchara PhumeeMichael GruschAtefeh ZarepourMichael H. BöhmeAthanasios KoukounarasMichael HässigAthanasios SamarasMichael J. KubaAthanassios AlexopoulosMichael J. VendrascoAthina NtzimaniMichael Jordan RowleyAtiah H. AlmalkiMichael KosoyAtif AdnanMichael LiebmanAtif AhmadMichael RustAtsuhiko YagishitaMichael SchwarzerAttila DénesMichael SeatonAttila ZsolnaiMichael W. BeckAtul Kumar PandeyMichał KalitaAudrey Opoku-AcheampongMichal KernAugusto VitaleMichał OczkowskiAulo ManinoMichal ŠtrosAurel Popa-WagnerMichal VágnerAurélien DeniaudMichal VinklerAurora ArghirMichał ZarobkiewiczAustin GraybealMichel AlmaguerAvi PeretzMichel BrondaniAvinash PremrajMichela IngrassiaAxel SteigerMichela PuglieseAyaka SatoMichele Christian KlymiukAyaz KhanMichele MarconciniAyaz ShahidMichelle GarciaAyca KocaagaMichelle LennartzAydin Bordbar-KhiabaniMichihiko AramakiAyman El-BadryMichio HidakaAyman Gamal Fawzy EL NagarMiguel Ángel Pérez-SousaAyse KoseMiguel Ángel PrietoAyslan Castro BrantMiguel Loera-SánchezAyush DograMihaela KavranAzize DemirpolatMihaela MureşanAzizur RahmanMihaela TurtoiAzza MostafaMikhail GantsevichAzzurra MargiottaMikko O. LaukkanenBabak NorooziMilad HadidiBabak PakbinMilad ShokriBabar HussainMilan KolářBabar ZahoorMilan TomaBabhrubahan RoyMilda ALksneBala Ani AkpinarMilena Álvarez-ViñasBalawant KumarMiles LangeBálint NagyMili DasBaocheng JinMilijana JanjusevicBaolin LiuMilos MatkovicBaoqian LyuMiłosz HuberBaosheng ZengMilva PepiBaoye HeMin GuanBarbara BertoglioMin Young LeeBarbara Di StefanoMing LiBarbara PietrzakMing XuBarbara WhitmanMingchong YangBarlin O. OlivaresMing-Fo HsuBarth W. WrightMingli LiBartłomiej NiespodzińskiMingxi ZhouBartosz BorczykMingyuan WangBartosz KruszewskiMinhyeok LeeBartosz TrzaskowskiMintu ChandraBashar IbrahimMinus Van BaalenBasílio A. M. GonçalvesMiquéias Lopes-PachecoBassam ElgamoudiMiquel PansBeata Guzow-KrzemińskaMira A. M. BehnamBéatrice GagnaireMireya Burgos-HernándezBeatrice Senn-IrletMiriam BelmakerBeatriz Buentello-VolanteMiriana D′AlessandroBehzad IravaniMirna MihelčićBekir KabasakalMiro JukićBelaghihalli N. GnaneshMirolyuba IlievaBelén RosadoMiroslav BarancikBen MansMiroslav BartákBence LázárMiroslava NedyalkovaBenedetta SaccomannoMisra VaruchaBeniamin Oskar GrabarekMitat KozBenoît BizimunguMitsuhiro FujinoBerca MihaiMizuho NishioBerceste GülerMladena Lalic-PopovicBernard PossidenteMohamad Mazen Hamoud-AghaBernardo VegaMohamed A. El-EsawiBernd MoosmannMohamed Al-SayeghBernhard ZdárskyMohamed MohanyBerta CsabaMohamed ShehataBerta HeinzmannMohamed SheteiwyBhaben ChowardharaMohamed TantawyBhakti TannaMohamed ZommitiBharani SrinivasanMohamed-Amine HassaniBhim Sen ThapaMohammad AkramiBhoomika PatelMohammad Al-HatamlehBianca Maria LombardoMohammad AltamimiBianka KarshikoffMohammad AslefallahBibhuti B. DasMohammad Belal HossainBibi KhanMohammad DelghandiBidisha RoyMohammad Faujul KabirBill J. TawilMohammad IrfanBin Abdul Rahim AzmanMohammad JamshidiBiplab PaulMohammad KamranBirgit GlasmacherMohammad Nurul Azim SikderBirgit MarcusMohammad Tavakkoli YarakiBirol AkbaşMohammad ZulkifliBiswajit MaharathiMohammadjavad MohammadiBlanca De-la-Cruz-TorresMohammed GagaouaBlanche CapelMohammed Shahidul AlamBo HuMohandoss SonaimuthuBo WangMohd SaeedBoguslawa LuzakMohd Yunus ShukorBonnie YoungMohit K. MidhaBono LučićMohsen M. RamadanBor Luen TangMohsen NiazianBora Florin DumitruMohsin KhurshidBorislav AngelovMohtadin HashemiBorja Cascales-MiñanaMoisés MarquinaBossima Ivan KouraMoises Roberto Vallejo PerezBozena DenisowMojie DuanBrahim SelmaouiMokded RabhiBrandon Lucke-WoldMolly AlbeckerBreda Jakovac-StrajnMolnár Tamás GergelyBrett MolonyMona C. GjessingBrian CrotherMona Van Schingen-KhanBrian D. WisendenMonica FrinchiBrian HanakMonica ProboBrice AutierMonika KoziełBrijesh M. ShahMonika Michałowska-SawczynBrijeshkumar PatelMonika TulejaBrindusa TiperciucMonish Ram MakenaBritt OpdebeeckMontse PérezBritta EggersMonwadee WonglapsuwanBrittany S. BarkerMor Nadia Lurie-WeinbergerBroder BrecklingMorales Nin BeatrizBruce BurnsMoses NyineBruna BatistaMostafa Ahmed AbooufBrunno CeroziMostafa OukassouBruno António CardosoMoustafa SarhanBruno FerreiraMuhammad AsadBruno José Nievas SorianoMuhammad AslamBruno PontesMuhammad ImranBryant H. KeirnsMuhammad JunaidBryce AsayMuhammad KhanBulbul AhmedMuhammad Moaaz AliBunushree BeheraMuhammad NumanBurkhard JakobMuhammad Sayyar KhanBushra ShahidaMuhammad Tariq JavedCaifei ZhangMuhammad UzairCallum George DonnellyMukesh MeenaCalogero Di BellaMukhum HammadCameron K. TebbiMukund BhandariCamilla BertoliniMukund V. DeshpandeCamille MoreauMumtaz AnwarCandelaria Martín-GonzálezMunazza KiranCanwei ShuMunetoshi MaedaCarl JohnsonMuntazir MushtaqCarla LuciniMurali MamidiCarla RagoneziMurray JelinskiCarla Sofia Sancho Dos SantosMustafa GhaziCarles BarriocanalMuthu SathishCarlo CinqueMuzamil Yaqub WantCarlo DiaferiaMyrna Leticia Bravo OlivasCarlo GualtieriMyron ChristodoulidesCarlo ReggianiN. BhagyaCarlo VascottoNa HuCarlo ZancanaroNabeel AhmedCarlos Alfonso Álvarez-GonzálezNabil RabhiCarlos Bueno-AlejoNadia BadolatiCarlos Díaz-CastilloNadia Maria SposiCarlos Eurico Dos Santos FernandesNadine BaumgartCarlos Felix MarinaNagah ArafatCarlos FigueiredoNagasuryaprasad KotikalapudiCarlos GoncalvesNai-Jen ChangCarlos GutiérrezNakamura AkihiroCárlos L. Ayán PérezNalinee TuntivanichCarlos M. ScavuzzoNaman VatsaCarlos MarcuelloNan BaiCarlos MassoneNana Y. AnkrahCarlos MedinaNanako KawaguchiCarlos O'Connor-ReinaNancy HelouCarlos Peña-GarayNancy OguiuraCarlos Pinto GomesNanette C. SchlootCarlos VicientNan-Jay SuCarlos ViegasNansi López-ValverdeCarmelo CorsaroNaoki HayashidaCarmelo Ortega RodríguezNaoto F. IshikawaCarmelo Peter BonsignoreNaoto YoshidaCarmen Daniela Quero CaleroNarendra Nath JhaCarmen Ortega-santosNaresh HanchateCarmen RizzoNaresh KumarCarmen SavinNaseem A. GaurCarmi KorineNaside MangirCarmine MarconeNatacha CoelhoCarol ClarkNatalí J. DelormeCarol WadhamNatalia Ivanovna AgalakovaCarolin PhilippNatalia KaczyńskaCarolina Alvarez MaldiniNatalia KasicaCarolina Castro SanguinoNatalia KoubassovaCarolina ChielliniNatalia MadetkoCarolina G. PoitevinNatalia MalloCarolina Gomez-DiazNatalia NaryzhnayaCaroline HastingsNatalia V. LebedevaCarolyn E. JonesNataliya GruntenkoCarsten HerskindNatalya A. ShnayderCassamo U. MussagyNatasha MaurmannCasto Juan-RecioNathalie GontierCataixa LópezNathalie PicaultCataldo PierriNathaniel HepowitCatarina EiraNathaniel SzewczykCaterina BonfiglioNathaniel T. BerryCaterina CiacciNawroz TahirCaterina RodríguezNazaruddin SinagaCathie PageNazli KhodayariCecile GouttefangeaNeamtu BogdanCecilia Alfonso-CorradoNeel K. PrabhuCecilia PaoliniNeeta MishraCecilia PerinNehaben A. GujaratiCecilia VeraciniNeil MabbottCélia GarciaNelly MohamedCéline SterNenad KoropanovskiCesar A. Rosales-NietoNeonila V. KononenkoCesar BordehoreNeptali Ramírez-MarcíalCesar Dionisio Jimenez RodriguezNerea Méndez-BarberoChairmandurai AravindrajaNereida M. Rancel-RodríguezChan Hee ParkNermeen Y. AbassChandani SenNese TuncelChandler C. BenjaminNestor Gutiérrez-MéndezChang LiNetta DorchinChang-Jun ZengNeven MatočecChao ShiNiall McGintyChao SongNicholas CrossChaofeng TuNicholas J. HaleyChaonan ZhangNicholas Marquez GrantChao-Yie YangNicholas PullenCharles MasonNicodemo G. PassalacquaCharles R. VossbrinckNicola CantasanoCharles VerneyNicola DecaroCharlie M. WaughNicola LambertiCharlotte ThomasNicola MondanelliCharlotte Von GallNicola SimoneChase H. SmithNicolae GicaChellappagounder ThangavelNicolas DumazChelsie CounsellNicolas GueroutChen Siang NgNicolas LaineChen ZhangNicolas PuillandreChenghao ChenNicolás TomasiniChengliang WangNicolas TurpinChenglin MoNicole ZiliottoCheng-Tao LinNicoleta UngureanuCheng-Yu LongNicoletta ContaldoChenjian GuNida AslamChenlin HuNikhil ChrungooChenyang CaiNikita ZelenkovCheuk Lun LiuNiklas TeliniusCheyenne TaitNikolaos SolomakosChi ZhaoNikolaos ZarasChia Shan WuNikolay A. BarashkovChian Ju JongNikolay G. BibikovChiara MagliozziNikolay PopgeorgievChiara Maria MottaNikos KrigasChiara PorroNils EkelundChiara VillaNimi VashiChih-Yang WangNina S. LilandChinedu Felix AmujiNinaad LasradoChing-Jin ChangNing ChenChinmay SatamNing ZhangChirag ParsaniaNino LäubliChi-Wei ChenNir SadeChoy Ker WoonNithya N. KuttyChris KourekNitin AmdareChris LoweNitin DhowlagharChrissoula VoidarouNivaldo T. SchieflerChristian Albrecht MayNoa NovershternChristian Frøkjær-JensenNobuhiro AburaiChristian ReynoldsNobuhiro SuzukiChristian SetzNobutaka SomeyaChristian VogeleyNoelia S. Bedoya-PeralesChristian WirajaNoor KazimChristian-François RoquesNor Rafeah TumianChristianna HowardNorhisham RaziChristina EmmanouilNoriaki MaedaChristina PacakNorifumi IijimaChristine BoneNorio YamamotoChristine ChappardNoritaka AdachiChristine ErbeNorman R. WilliamsChristine HertlerNounagnon AgbanglaChristine TachibanaNozomu OkinoChristoforos ThomasNunzio Antonio CacciolaChristoph FrauneNunzio VicarioChristoph FriedliNur ‘Aliah DaudChristophe BattailNurul Yuziana Mohd YusofChristophe BilletteNuttapon PombubpaChristophe BressacNuttawut SermsathanasawadiChristophe DomingosNuzhat ShafiChristophe HanoNycolas Levy-PereiraChristopher A. BeaudoinOadi MatnyChristopher B. BoykoOana Cristina ŞeremetChristopher BallmannOctavian Tudorel OlaruChristopher BertramOctavian-Gheorghe DuliuChristopher ClealOdalisca BreedyChristopher CockelOfir KatzChristopher J. BellOgueri NwaiwuChristopher J. ClealØivind BerghChristopher P. ToselandOkio HinoChristopher R. WestOlaf GriskChristopher RobbinsOlav YriChristopher Robert BridgesOleg YakhinChrysoula BetsouOlga DolgovaChrysoula G. OrfanidouOlga EzhovaChryssanthi AntoniadouOlga GavrichkovaChuan LiuOlga Jovanovic GlavasChuanyu YangOlga Nicole Kokiko-CochranChun LiOlga PosukhChunui LeeOlga S. SamylinaCindy KörnerOlga S. TarasovaCingel AleksandarOlga SelyutinaCinta ZapaterOlga V. EfremenkovaCinzia MasperoOlga V. FedorovaCiro CelsaOlga V. RazumovaClaire AllenOliver D. TavabieClara F. RodriguesOlivera DjuragicClarissa M. BruscoOlivia A. KimClarisse BrígidoOlivier DutourClaudia CantoniOlivier GlaizotClaudia CrociniOlivier JaillonClaudia Daniele Carvalho NavarroOlivier Tremblay-SavardClaudia DittfeldOliviero CarugoClaudia DurantiOmayma MissawiClaudia GiannettoÖmer Faruk BayrakClaudia MatthäusOndrej BonczekClaudia Mettke-HofmannOPhir NaveClaus MosekeOrhan Tansel KorkmazClaus VoglOrhan YenigunClayton J. HilmertOri PomerantzClifton HolmesOriana TrubianiClifton P. Bueno De MesquitaOriol Abellan-AynesColin GatesOriol De BarriosConcepció MarinOscar Martinez De QuelConcetta Di NoraÓscar Rapado-GonzálezConstance CiaudoOscar Sosa-NishizakiContreras-Medina RaúlOskar A. PalaciosCorina Marilena CristacheOskar SiemianowskiCornelius KraselÖzgür KuzukıranCornelya KlutschÖzlem ÖzmenCorrado PaganelliP. P. SujithCorrado PiconiPaapa DasariCorrado SantocanalePablo Arechavala-LopezCosmin EnePablo CastilloCostanza JuckerPablo Fernández-TussyCrisalejandra Rivera-PérezPablo Luna-NevarezCristal ZunigaPablo Valdés-BadillaCristi L. GalindoPajor FerencCristian DelceaPalak PatelCristián JacobPalaniappan AshokCristian PetriPaloma Guillem-LlobatCristian StătescuPamela TozzoCristina Burrola-AguilarPanagiota XaplanteriCristina CasalsPanagiotis EfentakisCristina CozziPanagiotis F. LiaparinosCristina EnăşcuţăPanagiotis GianniosCristina Meniz GarciaPanagiotis GkriliasCristina NadaluttiPanagiotis KatinakisCristina SecosanPanagiotis Nikolaou MoschouCristina Tomás-AlmenarPanagiotis TsimeasCristina VercelliPanayiotis LoukopoulosCristobal EspinosaPandisamy RagavanCristoforo PomaraPaola BraghettaCrystal S. ShinPaola NavarreteCuauhtémoc Sáenz-RomeroPaola TedeschiDaiki TakahashiPaolo AbondioDailu GuanPaolo ConflittiDaisuke MiyoshiPaolo DapavoDaitoku SakamuroPaolo FagoneDamien CunyPaolo GrittiDamir ZubacPaolo MartelliDan KeremPaolo PascoloDan Mihai RohozneanuPaolo RosaDan QiPapale MariaDana BadauParaskevi LampropoulouDana J. HolgerParimal SamirDana Lucia StanculeanuParthasaradhireddy TanguturiDana UlanovaPascal PineauDaniel A. MedinaPasquale MoneDaniel BarbosaPatricia Agudelo-RomeroDaniel G. PetersonPatricia GodoyDaniel MeltersPatricia Gómez-VillegasDaniel P. PotaczekPatrícia Oliveira FiuzaDaniel P. ZalewskiPatricio De Los Rios-EscalanteDaniel PotterPatricio Orellana-PalmaDaniel Reis WaisbergPatrick AnglardDaniel RodriguezPatrick CadetDaniel RoethPatrick CelieDaniel SedorkoPatrick J. KrugDaniel Tsun-Yee ChiuPatrick Mark SmithDaniel W. NixonPatrick TéouleDániel WinklerPatrizia FalabellaDaniela Cajado-CarvalhoPatrycja KleczkowskaDaniela GabbiaPatrycja SchulzDaniela IsolaPau Redón LurbeDaniela Silvia PacePaul A. SaundersDaniela TakacovaPaul F. WilliamsDaniela TrabattoniPaul SheppardDaniele CafollaPaula AranazDaniele De SantisPaula Esteban-GarcíaDaniele MercatelliPaula Karine JorgeDaniele PironiPaula Ramos-SilvaDanijela SpasicPaula VissioDanilo MarimpietriPaula Witt-EnderbyDanis AbroukPaul-Alexandru PopescuDanny LumPaulina AdamskaDaomin PengPauline VannierDaoying GengPaulo A. HartmannDaoyuan ZhangPaulo A. V. BorgesDario Di SilvestrePaulo MartinsDariusz KucharczykPaulo MascarenhasDariusz KulusPavan Kumar DhanyamrajuDariusz PawlakPavel I. KostylevDarja BožičPavel KotrbaDarko BosnakovskiPavel NekhoroshkovDarlene MitranoPavel SolopovDaryl BowmanPavel ZelenikhinDavid B. CroftPavol DubinskyDavid BouffordPavol ZelinaDavid CatelaPaweł BuczyńskiDavid ChelazziPaweł KowalczykDavid ColePaweł LechDavid DeShazerPaweł PietkiewiczDavid DraperPaweł ŚliwaDavid J. MathewPayam BehzadiDavid LaightPedro A. Jiménez GómezDavid LawPedro CisternaDavid LindenmayerPedro José Tauler RieraDavid M. LehmannPedro L. CastroDavid MartínPedro Luis Ramos-GonzalezDavid Medina-CruzPedro MorouçoDavid MerrittPedro NavesDavid MorgensternPedro PellicerDavid OharaPedro PiñeroDavid P. MallonPedro Valdivia-MoralDavid R. BurgessPei Yuin KengDavid S. NavegaPeichao ChenDavid ŠilhaPei-Gen RenDavid StankovićPeipei ZhangDavid Viejo-RomeroPejman RohaniDavid W. OwensPeng ZhaoDavid ZweikerPengbiao XuDavide AsnicarPengda LiuDavide CavagnettoPengfei HanDavide GuerraPengze YanDavide SoglianiPere M. Parés-CasanovaDawid StormanPerla Jannet Jurado-GarcíaDearbhaile DooleyPero MijićDebajyoti BosePerry B. HackettDebmalya BarhPerveen SummiaDebora ZurroPetar ĐanićDebra A. WillardPetca Razvan-CosminDeepak KumarPeter A. HarrisonDeepanwita BanerjeePeter A. HendersonDeepika SinglaPeter A. RoussosDehao FuPeter AskjaerDehua WangPeter CakebreadDeividas ValiunasPeter ConveyDelio José MoraPeter GronesDelphine ThibaultPeter JohnstonDemetrio LozanoPeter LacknerDengqiu LiPeter McGranaghanDenise DostatnyPeter MylerDenise Paschoal SoaresPeter PutzDenise SchramaPeter R. LaityDeniz ErgudenPeter SmithamDeniz F. ErezyilmazPeter SteinbacherDeniz InnalPeter ValloDennis GreerPēteris TretjakovsDennis HeinrichPethaiah GunasekaranDeona BothaPetr BahenskýDhananjay YadavPetr KocarekDhara SharmaPetr ŠkorpíkDharmeshkumar PatelPetr TvrdikDi WuPetra FrýdlováDiana Hernandez-RomeroPetra KameritschDiana López-AlvarezPetranović Matea ZajcDiana Marcela CastilloPetro TsarenkoDiana NicaPhil HandcockDiana O. TreabaPhilip RenoDiana RabadjievaPhilip ZegermanDiana Rodríguez-HurtadoPhilippe FavreDiana SilvaPhilippe RobertDiana TonevaPhillip D. WhitfieldDiana Vasca ZamfirPhillip DettleffDiana ŽaliaduonytėPhil-Robin TepasseDiego Franco JaimePier Nicola SergiDiego NogueiraPier SaccoDiego TesauroPiera Anna MartinoDigant NayakPiero CossuDilek MercanPierpaolo AlongiDimitra Ermione MichaelPierre-Guy MarnetDimitri KakabadsePierre-Simon JoukDimitrije MarkovicPieter De LangeDimitrios A. AnagnostopoulosPietro BarbacciaDimitrios KaragiannakisPilar BlancoDimitrios N. AvtzisPınar CihanDimitrios SklirosPing FangDimitris GrammatopoulosPinuccia FavianaDimitris TigkasPiotr ArtiemjewDimitris TsakogiannisPiotr DobryszyckiDimitry SchepetovPiotr SzwedaDinu GavojdianPiotr WlaźDiogo Luís MarquesPiyush BaindaraDipsikha BiswasPolosa Paola LoguercioDiptee ChaulagainPoonam PhalakDirk GeertsPornthap ThanonkeoDirk MetzlerPoulami SarkarDirk MontagPrabhu RamamoorthyDirk TischlerPrabu GnanasekaranDivya DasagrandhiPradeep CingaramDmitri KramerovPradeep Kumar SudheeranDmitri SobolevPradeep SinghDmitry B. ZorovPradeepa SilvaDmitry NikolayevPragathi Belagola ShridharDobrochna Adamek-UrbańskaPragneya DemeDobromira ShopovaPrakash BhuyarDomenica FarciPrakash SahDomenico IannelliPramesh SinghDomenico LioPramod ShindeDomenico ScaramozzinoPrashant JainDominic Paquin-ProulxPrashant KumarDominika GłąbskaPrasun K. DattaDon GreenPrateek LohiaDonatas ŠneiderisPrateep PanyadeeDonatella Degl′innocentiPrathap SomuDong Wook ShinPratiti BhadraDong-Hyun ShinPraveen PandeyDong-Kyu KimPravin KaldhoneDong-Xiu XuePrem Pratap SinghDora Aguín-PomboPriscilla AmaralDóra ReglödiPriti GuptaDoris Rosero-GarciaPrzemysław DomaszewskiDorota HilszczańskaPrzemysław KołodziejDorota WojniczPrzemyslaw KowalDorota WultańskaPrzemysław SobiechDouglas Grant OsbornePuneet BatraDouglas S. GlazierPurna PutraDragan MarinkovicQaisar MahmoodDragan MilicevicQazi Mohammad Sajid JamalDragoljub GajicQi FangDuarte BarralQi ZhangDuarte Henriques-NetoQihao WuDung Ha-TranQinghua LiuDurai SellegounderQingsen ShangDusan MisicQingwen ZhangDuy Ngoc DoQinyu SunDyah Kurniawati AgustikaQinzhe WangDzarifah ZulperiQiong ZhangEbenezer Antwi GyameraQiongxian YanEberhard UhlQiusheng YuanEbru YıldırımQiuwang ZhangEdeline GagnonQixiang LinEdgar LehrQuan ZhongEdgar Rodríguez-NegreteQutaiba AbabnehEdiane ZaninRachael CarewEdit É. FarkasRachel L. WashburnEdmund Ilimoan YambaRachel Roberts-GalbraithEdmundo Bonilla GonzálezRachelle MendozaEdna Sadayo Miazato IwamuraRadhia M′kacherEdo D′AgaroRadim VáchaEdoardo MuratoreRadojica DjokovicEdouard GerbaudRadomir SavićEdson TalaminiRadoslav BeňušEduardo Antonio SperanzaRadoslava KralevaEduardo BarrónRadoslaw LenarczykEduardo BoidoRadoslaw PuchalkaEduardo Garcia-RicoRadu BotgrosEduardo GonzálezRadu TamaianEduardo José Rodríguez-RodríguezRafael Carrasco-ReinadoEdward A. LawsRafael De AlmeidaEdward PavlikRafael Gutiérrez-LópezEdward QuadrosRafael J. BorgesEdward R. SauterRafael Julio Macedo BarragánEdwin CadenaRafael Lahoz-BeltraEfstratios-Stylianos PyrgelisRafael OliveiraEfthymia NikitaRafael Prado-GotorEhab El-HarounRafael Rodrigues DihlEhsan AhmadpourRafael Rodríguez MartínezEhsan KheradpezhouhRafael Salto-GonzalezEhsanul Hoque ApuRafael Victorio GuidoEileen A. HebetsRafał LasotaEirini Kanella PanagiotopoulouRafał SapierzyńskiEitoyo KokubuRafał StarzyńskiEkaterina A. ZelentsovaRafał SzafraniecEkaterina V. ZubarevaRaffaele PalmirottaEkrem OzluRaffaele SerraEladl EltanahyRaffaella BelvedereElaheh Dalir AbdolahiniaRaffaella CascellaElaine EmmersonRaheleh RoudiEléanor LuceRahul BhowmickEleftherios TouloupakisRahul ChandnaniElena Elena BarcanuRahul GauttamElena F. ShevtsovaRahul Kumar SinghElena FrigatoRahul NelliElena KraniotiRai Khalid FarooqElena Labajo GonzálezRaimondas MozuraitisElena López-GallegoRaimund WidhalmElena Martinez-SanzRajbir SinghElena MontiRajeev PadbhushanElena S. BelykhRajeev YadavElena V. SabaneyevaRajesh AngireddyElena Valeria FuiorRajesh KomatiElena Villar-NavarroRajesh KumarEleni GolomazouRaju MurugananthkumarEleni KarligiotouRamasatyaveni GeesalaEleni NintouRamendra Pati PandeyEleni VerykoukiRamesh PeriasamyEleonora CappellettiRamon MalheirosEleonóra SpekkerRamya BillurEleuterio SánchezRamzi MahmoudiElfatih M. Abdel-RahmanRando TuvikeneElia GattoRanjan RamasamyElia RosiRaphael Igor DiasElina Yankova-TsvetkovaRaquel De Cássia Dos SantosElio RomanoRaquel Lama LópezElis NewhamRaquel Leirós-RodríguezElisa Lopez-DoladoRaquel Sanabria-De-La-TorreElisa ZavattaroRasha GhariebElisabeth BlüthnerRashad AlfkeyElisabetta AffabrisRashmi AkulaElisabetta BaldiRashmi YadavElisabetta BuomminoRasmus GustafssonElisabetta RazzuoliRatna GhosalElisavet KaitetzidouRatnakar DeoleElison Fabricio Bezerra LimaRavi Chand BollineniElitsa DeliverskaRavi Kumar KomaravoluElizabeth CordobaRavikumar PatelElizabeth HenryRavindra DeshpandeElizabeth HillRavindra PeravaliElizabeth Noemi LlanosRavindra ThakkarEllederová ZdenkaRay CorrellElmira MohandesanRay KuElodie MagnanouRaymundo Buenrostro-MariscalElpiniki SkoufogianniRazvan BocuElsayed MetwallyRebecca AlexanderElżbieta PatkowskaRebecca C. HenryElżbieta SpeinaRebecca CreamerElżbieta WyskaRebecca PanconesiEmami Bistgani ZohrehRebecca RolfeEman MohallalRebecca TaylorEman ZahranRebekah R. HeltonEmanuel BaltagRedha TaiarEmanuela FanelliRedi RahmaniEmanuele BernardinelliRekha JagadapillaiEmanuele Del GuacchioRémond YvesEmiliana GiacomelloRenalison Farias-PereiraEmiliano BediniRenata Del CarratoreEmiliano CèRenata SoutoEmilie Réalis-DoyelleRénato FroidevauxEmilie WidemannRené Massimiliano MarsanoEmilio CervantesRen-Jay SheiEmilio J. GualdaReza MazaheriEmilio ParisiniRhonan Ferreira Da SilvaEmilio V. Carral VilariñoRicardo LucenaEmily R. RyznarRicardo Nalda-MolinaEmma PeelRicardo Pérez-de La FuenteEmmanouil MalandrakisRicardo R. González-MéndezEmmanuel AkindeleRiccardo BrunaEmmanuel Navarro-FloresRiccardo MorettiEmmanuele CrespanRiccardo ProiettiEmyr PeñaRiccardo VioEng Lee OoiRichard A. AmosEng Soo YapRichard D. FoustEnquan XuRichard H. SteckelEnrica RosatoRichard HenriksenEnrica SantucciRichard IvellEnrico D′AnielloRichard O′RorkeEnrico DoriaRichard W. BohannonEnrico Fuini PugginaRicheng ChianEnrico PerrinoRicki ColmanEnrico RuizRicky LaraEnrique Domínguez-ÁlvarezRie Roselyne YotsuEnzo GorettiRikke BirkedalEphraim ParentRima LucardiErden AtillaRisely Ferraz AlmeidaErez Bar-HaimRishi NageshanErgun KayaRisma I. MaulanyEric C. BeyerRita CordeiroEric DarrouzetRita LorenziniEric M. SmallRita MarulloEric S. HoRita SorrentinoErich SchwarzRiu YamashitaEricka L′AbbéRobert AncuceanuErik LindRobert BodeÉrika Said Abu EgalRobert ĆelićErin RehrigRobert E. LarsonEsaú Ruiz-SánchezRobert GałązkowskiEsengül ÖzdemirRobert GrabarczykEslam M. Abdel-SalamRobert J. WalkerEsmaeili Hamid RezaRobert KasprzakEsmat AliRobert KoflerEstanis NavarroRobert NofemelaEstefania Martinez TaveraRobert T. BradyEstève BoutaudRobert T. MeansEsther IsornaRobert TrigianoEstibaliz CastilleroRobert W. A. PottsEszter KapusiRobert W. ElwoodEszter Patakiné VárkonyiRobert Walter MannEtienne SchwobRoberta AndreoliEugénia CunhaRoberta LottiEugenio JannelliRoberta PisciaEugenio ProvenzanoRoberta TittarelliEun Jeong ParkRobertas DamaševičiusEun Kyung LeeRoberto BaroneEunsuk KimRoberto CaldaraEustachio TarascoRoberto Franco Enrico PedrettiEva KudovaRoberto GramignoliEva María Martínez-JiménezRoberto LandeEva MrkvicovaRoberto MantovaniEvan Yi-Wen YuRoberto Martínez BeamonteEvangelia KerezoudiRoberto Oliveira DantasEvangelia SmetiRoberto VoglerEvangelos G. KotsonasRobin Lewis CooperEvgenia Korzhikova-VlakhRobin NicholasEvgeniy S. BalakirevRocio Eiros BachillerEvgeny A. BorovichevRoderjan João GabrielEwa Joanna GodzińskaRodney Alan BrayEwa RopelewskaRodolfo C. GarciaEwa WasilewskaRodolfo GentiliEwa Zabłocka-GodlewskaRodolfo GozaloEwan ThomasRodolfo Ramos GonzálezEwelina MaculewiczRodrigo GigliotiEwelina Warzych-PlejerRodrigo Gomes Da RosaEwoud B. CompeerRodrigo MonteiroEyal SeroussiRodrigo Moore-CarrascoFabian KanzRodrigo OkuboFabian WeykampRodrigo Petry Corrêa De SousaFabiana CaniniRodrigo VidalFabiana CrispoRodrigo ZaccaFabiana GeraciRogério Leone BuchaimFabio CianferoniRohit KumarFabio CiccaroneRoland MelzerFabio Del DucaRoland StauberFabio F. NocitoRolf BarthFábio FernandesRomain GuinamardFabio GuoloRoman CroitorFabio MarroniRoman S. NagovitsynFabio Matos ChiarelliRomana MoutelíkováFabio OstiRomana VulturarFabio StochRomeo Petru DobrinFahad KhanRomina S. PetrighFahd RasulRômulo AlvesFahimeh VarzidehRonald B. BrownFaihanna Ching AbdullahRoohallah Saberi-RisehFakhredin SabaRosa BellavitaFaming WangRosa Colucci CanteFan LinRosa Di CrescenzoFan ZhangRosa LinaresFang Rong HsuRosa VallettaFangyu LiuRosalia CrupiFani NtanaRosanna PaolinoFanju MengRosario García-GiménezFarzad KianersiRosilda Mara MussuryFarzam TavankarRossana IzzettiFathy ElbehiryRossella MieleFatima AwwadRovshan KhalilovFatima MucceeRoxana Adriana StoicaFatima RivasRoxana LucaciuFausto Machado‐SilvaRuan VeldtmanFausto MaffiniRubens T. D. DuarteFebina MathewRuby DawsonFederica FogacciRudian ZhangFederica GuffantiRüdiger MaschkeFederica MagagnoliRui XuFederica MontesantoRui YangFederica ValerianiRuifeng HuFederico FontanaRuimin QiaoFederico Rivas FrancoRuiying ZhaoFei ChengRukman Awang HamatFei FangRulin ZhuangFei YeRumesh RanjanFelipe Contreras-BriceñoRupesh DeshmukhFelipe José AidarRusan CatarFelipe PezoRusănescu Carmen OtiliaFelipe SarmientoRuslan KalendarFelix G. SauerRussell D′SouzaFélix Javier Jiménez-JiménezRustem IlyasovFeng ChengRuud G. J. MeulenbroekFeng CuiRuwei LinFeng WangRyan MaysFeng XueRyan S. PralleFeng-Hua SunRyan SamuelFengqing YangRyo NozuFerdinando IellamoRyota HosomiFerenc A. AntoniRyszard GrendaFernanda AchimónRyuichi FukudaFernanda GentilS. M. Shatil ShahriarFernando Barroso-BarcenillaSaba Ahmed BeighFernando SantosSaba BattelinoFernando VonhoffSabino PachecoFilip MilanovicSabrin IbrahimFilipa JoãoSabrina DemarieFilipa MendesSabrina Di BartolomeoFilipa R. PintoSabrina OlivaFilipe M. CunhaSabrina Rahman ArchieFilipe Manuel ClementeSachi Nandan MohantyFischer HillarySadatomo HisamatsuFlamur SopajSae TanakaFlavia GiamoganteSaeid KargozarFlorentina PluteanuSagar MaitraFlorian SchachingerSage ChaiyapecharaFloyd W. WeckerlySahil MehtaFogang AymarSai Shiva Krishna Prasad VurukondaFortunato FernandaSaïd MouzeyarFotis E. PsomopoulosSaima RubabFouad DabboussiSajjad AhmadFouquet GuillemetteSakshi RajoriaFranca AnglaniSalihu IbrahimFrances Meier-GibbonsSally Ann HuberFrancesc Fusté-FornéSalvador MireteFrancesca Boscolo SesilloSalvador PérezFrancesca De FalcoSalvador Romero-ArenasFrancesca GrippiSalvatore NesciFrancesca LatinoSalvatore ScolavinoFrancesca Romana FuscoSamantha KarunarathnaFrancesca VenturaSamarendra DasFrancesca ZitoSameer Ahmed AwadFrancesco CanonicoSameh SelimFrancesco CiarleglioSami Abou FayssalFrancesco Di MolaSamira El OtmaniFrancesco FazioSamuel Abalde LagoFrancesco FischettiSamy M. SayedFrancesco M. AngeliciSanaullah SajibFrancesco MaffìaSandeep SharmaFrancesco NardiSándor BarthaFrancesco StiloSandra Agius DarmaninFrancesco TarantiniSandra CellinaFrancesco ZangaroSandra Cruz MarcosFrancisca Alves CardosoSandra EčimovićFrancisca Reyes-RamírezSandra HudinaFrancisco A. ArrebolaSandra HuérfanoFrancisco A. MartínSandra L. Tecklenburg-LundFrancisco Callejas-HernándezSandra L. WellerFrancisco CastilloSandra McFaddenFrancisco CurateSandra PucciarelliFrancisco E. NicolasSandra RodriguesFrancisco FernándezSanford I. BernsteinFrancisco GodinhoSang-Ha KimFrancisco J. López-BaenaSanghoon KangFrancisco J. Pérez-GarcíaSang-Wook ParkFrancisco J. Vega-VeraSanja TomšićFrancisco Javier Pérez-BarberíaSanjay SainiFrancisco Otero-FerrerSanjucta DuttaFranco ArturiSanna OlssonFranco MutinelliSanne Simone KaalundFrançois LalondeSantiago CepedaFrank KrauseSantiago GrijalvoFrank WyattSantiago MoraFrédéric MarsolaisSantiago Pineda-MetzFrederik GrawSantiago ZabaloyFriedrich LadichSantidan BiswasFuat Hakan SanerSantosh K. YadavFugo TakasuSantosh KumarFulgencio AlatorreSantosh UpadhyayFulin LuoSaowaluck TibprommaFulong YuSapna LangyanFulvio CelsiSara BerganteFulvio D′AcquistoSara BernardiFulvio PupilliSara CarmoFumin WangSara FaggionFumio YokohariSara FerrandoFurong XuSara GodenaGábor BotfalvaiSara L. BushmannGábor MátisSara R. NoumeurGábor SomlyaiSara RemelliGabriel Claudiu TenderSara Rodríguez-AcebesGabriel Dias RodriguesSara Sánchez-MorenoGabriel González-ValeroSarah Ann WoodinGabriela-Dumitrita StanciuSarah CuryGabriele MascheriniSarah MazzottaGabriele MulliriSarah NaiyerGabriella GulyasSarbani GhoshalGabrielle C. MuskSarfraz ShafiqGaelle Guiraudie-CaprazSarmila TandukarGaetano CataneseSasivimon SwangpolGaetano Magnano Di San LioSathya VelmuruganGaetano SantulliSatish RainaGagandeep SinghSatoshi KatayamaGail AndersonSatoshi YuguchiGalal MetwallySaturnino Marco LupiGaldino AndradeSatya Eswari JujjavarapuGalina A. ZhouravlevaSatyabrata NandaGalina V. KopylovaSaúl Martín RodríguezGang FuSaumik PanjaGaohua JiSaurabh MishraGary GaoSaurabh PandeyGary Hin-Fai YamSaurabh SinghGary MaSavvas LampridisGary StringerSayed Haidar Abbas RazaGáspárdy AndrásScott D. LundyGaston A. Bazzino FerreriScott D. RolosonGavriil George ArsoniadisScott W. TalpeyGe ZhouSean D. TallmanGebremariam BirhanuSebastian EscobarGelu OnoseSebastian GisderGema Barrientos VichoSebastian J. SchultheissGenhua NiuSebastian UlrichGeoffrey H. SperberSébastien AlfonsoGeorge E. MustoeSébastien JaquemetGeorge FthenakisSébastien PenninckxGeorge KraemerSedighe KarimzadehGeorge MichailSedigheh DelmaghaniGeorge N. HotosSeiji InoueGeorge P. LomonossoffSeiji TanakaGeorgia BraliouSeinen ChowGeorgia KarpathiouSeiya OuraGeorgiana NitulescuSelma Maria Almeida-SantosGeorgios DiamantopoulosSenem KamilogluGeorgios GkafasSenta NiedereggerGeorgios K. MarkantesSenthilnathan LakshmananGeorgios LaliotisSeong-Hee Maria KoGeorgios PapadopoulosSerdar BabacanGeorgios S. AmiridisSerena BoninGeovanny BarrosoSerena DattolaGeraldine GononSerge V. NaugolnykhGeraldine Zimmer-BenschSergei Andreevich DzubaGerard J. M. VerkleySergei F. ChekmarevGerardo Romo-CardenasSergei TuranovGerhard MoitziSergey MoskvinGermán M. López-IborraSergey SedykhGerrit BegemannSergey ShekhovtsovGéza RipkaSergey Yurievich StorozhenkoGhazi RacilSergii KrysenkoGholamreza HassanzadehSergio AcínGiacomo GattoniSergio Gastón CaspeGian Luca MarellaSergio GhidiniGiancarlo CondelloSérgio SilvaGiancarlo CuadraSeung Kyu LeeGianfranco PiconeSeung-Joon AhnGianluca RizzoSevdan YilmazGiannis D. ParaskevopoulosSeverino Jefferson Ribeiro Da SilvaGidon EshelSeyed Mehdi RajaeiGilbert Nchongboh ChofongSeyed Mohammad Ali Hosseini RadGilles BreuzardSeyit Ahmet ErolGilles GargalaSeyyed HoseiniGilmário Ricarte BatistaSezai ErcisliGina Delia Roque-TorresShafat AliGinevra BoldrocchiShah FahadGioacchino BonoShahabaldin RezaniaGioele CapilloShahid FarooqGiora KidronShahinul IslamGiorgia GiordaniShailaja Kesaraju AllaniGiorgio F. GilestroShailesh KumarGiovanna PotenzaShakhawath HossainGiovanni GalloShalini K. SharmaGiovanni PalleschiShamsaldeen Ibrahim SaeedGiovanni TarantinoShan BianGisèle LaPointeShane AustinGisella VetereShangru LyuGiselle YeoShangzhe XieGiulia BosioShannon CrowleyGiulia FranzoniShannon L. CartwrightGiulia GastaldiShanti GangwarGiulia GiuntiShaohua ChenGiulia RioloShaopeng PeiGiulia RonchiSharafaldin Al-MusawiGiulio MarchesiniSharareh SharififarGiuseppe ApreaSharat PradhanGiuseppe BanfiSharath GaddelapatiGiuseppe CalcagnoShari CohenGiuseppe CaminitiShashi Shekhar SinghGiuseppe De MarcoShaun LeiversGiuseppe EspositoShawnda A. MorrisonGiuseppe G. LoscoccoShengkun XieGiuseppe MalfaSheng-Nan WuGiuseppe Murdaca‪Shengqian XiaGiuseppe MusumeciSheng-Shan LuGiuseppe RemuzziSherif RashadGiuseppe SignorielloShiang-Jong TzengGiuseppina AndreottiShigehiko OgohGiuseppina MartellaShigeki MatsubaraGiusi MacalusoShigenori TanakaGizeh LazaroShigeru WatanabeGlayciane Costa GoisShih-Hsun HungGlen PyleShihuo LiuGlenn P. DorsamShih-Yi PengGloria Álvarez-SolaShijie HeGloria MazzoneShile HuangGodelieve GheysenShimaa AmerGökhan KarakülahShinichiro OgawaGomes CátiaShining LooGonzalo G. BarberáShinya ImadaGonzalo Nieto FelinerShiva Theja Reddy SomaGopinath KrishnasamyShivangi InamdarGoran KlobucarShivankar AgrawalGoran KrstacicShi-Yong MengGoran PalijanShizhao LiGordana Blagojević ZagoracShizuo KatamotoGordon Turner-WalkerShogo SatoGorji MarzbanShowkat DarGösta EggertsenShray SaxenaGouranga BiswasShu Wen WenGoutam SarkerShuai RenGrace FarhatShubha Ranjan DuttaGraciela Catalina Alatorre-CruzShuchen FengGraciela GarcíaShuguang WangGraham MartinShuo SuiGraham SmithShu-Ping WuGraziano FioritoShuqi LiGrażyna JanikowskaShuvasree SenguptaGregorio GarciaShweta KukrejaGregório Miguel Ferreira De CamargoShymaa EnanyGregorio VonoSibel Alagoz ErgudenGregorius Satia BudhiSiddhaling UrolaginGregory BarshteinSiddharthan SelvarajGregory BuckSifat MomenGregory JohnsonSigal NaimGregory P. BartonSijin GuoGrzegorz JanuszSilvano FerriniGrzegorz NowickiSilvestre Bongiovanni AbelGuadalupe Rojas-SanchezSilvia Alejandra García-GascaGuan-Da SyuSilvia BoladoGuang YangSilvia D′AgostinoGuangsheng YuanSilvia GutierreGuan-Jhong HuangSilvia LiskovaGuglielmo DurantiSilvia Maria MarcheseGuido PietroluongoSilvia Marquina-BahenaGuido ValenteSílvia Rocha-RodriguesGuihong BiSilvia Stefania LongoniGuillaume BilodeauSilvina LompardíaGuillermo PlazaSilvio Assis De Oliveira-JuniorGulasekaran RajaguruSima SinghGünter GollmannSime VersicGünther GerischSimon A. MorleyGuo ChenSimon LyttonGuojun WuSimona GaberščekGuosheng SuSimona MaccheriniGuruprasad MahadevaiahSimona MoldovanuGustavo AmoresSimone BonamanoGustavo De Carvalho-SouzaSimone BrogiGustavo Desire Antunes GastalSimone CantamessaGustavo HeidenSimone FilardoGustavo SantoyoSimone GalloGuy BeauchampSimone MorselliGyörgy SiposSimone Riis PorsborgH. Michael G. LattorffSimone RossiHabib KhanSimonida TomićHaewon ByeonSirijan SantajitHafeez Ur RahimSiva Karthik VaranasiHafiz Ishfaq AhmadSivaraman NatarajanHafiz Tayyab RaufSiyi GuHaitao WangSladjana JevremovicHaitham JahramiSlobodan DavidovićHajime YaegashiSmriti SinghHakan BozdoğanSneha SitaramanHakan MaydaSneha VivekanandhanHalil İbrahim CeylanSnezana StankovicHalil YildizSofia GamitoHamada ElwanSofia Villacis-NunezHamdi ChtourouSofiene Ben KaabHamed MozaffariSoha AhmadiHamid Reza MaratebSohail AhmadHammad A. GanatraSohan JheetaHana BandouchovaSohei KobayashiHana M. DobrovolnySoibam Khogen SinghHani Ba-AwadhSokhee JungHan-Jia LinSolomon AfelikHanna BjörckSom DevHanna HuebnerSomenath ChakrabortyHannah WuSompong ChankaewHans ReinkeSoňa ČiernikováHany M. R. Abdel-LatifSonam MittalHao GuoSong HeHao LiSongli ZhuHao MaSongül ÇakmakçıHao NieSonia BenitezHao ZhangSonia Brito-CostaHarald BeckSonia LaneriHarald LahmSonia ScarfìHarikumar RajaguruSonja SaloviusHarilaos A. LessiosSophia M. SchmitzHarold J. G. MeijerSophie VincentHarold P. EricksonSorana MateiHarold SchreierSoraya Paz MontelongoHarpal SinghSoshi SamejimaHarshani WijerathneSotirios ApostolakisHasan MehrajSotiris AmillisHaslina TaibSouhail HermassiHayato TerayamaSoumya IyerHayato TsukamotoSouvick RoyHaydeé Rosas-VargasSowmya PoosapatiHayford ManuSowmyalakshmi SubramanianHayley GlassicSpyridon PapageorgiouHeather GarvinSrba MijailovichHeather WilkinsSrdjan MarkovicHeba A. GadSreenivasulu ChintalaHéctor Capella-MonsonísSreeram UdayanHector HerreraSrijani BasuHéctor RuizStan TrauthHeidi KluessStanislav LazopuloHeikki MinnStanislav TrdanHeitor CorrêaStanisław HorakHélcio Gil-SantanaŞtefan Gregore CiorneiHelena CastroStefan Marian FrentHelena FelgueirasStefan WoelflHelena KorpelainenStefania ToselliHelena LenasiStefanie KleinHelena RyšlaváStefano AmatoriHelene RimeStefano BarbiHeloisa Sobreiro Selistre De AraujoStefano FilacordaHenner BrinkmannStefano NobileHenning SørumStefano PaganoHenrik KoblauchStefano ZiccardiHenrique QueirogaStefanos AndreadisHenrique Rocha MendonçaStefanos LeontopoulosHenrique SilvaStepan AleshinHerbert Ryan MariniStepan GambaryanHermansyah HermansyahStephan HeisingerHernández HerreroStephan Joel ReshkinHernando P. BacosaStephan KerstingHervé ReychlerStephan KoblmüllerHidekazu HiroakiStephanie GamezHideo KatoStephanie ParkerHideo SuzukiStephen A. WankHideo WadaStephen WintersHideyuki MaedaStephen YarwoodHiginio López-SánchezSteve SchapiroHilton M. OyamaguchiSteve SuhHimadri Sekhar SarkarSteven F. GameiroHiroetsu SuzukiSteven M. PascalHiroki NakaharaSteven T. LeachHiroshi AraiStevo PopovicHiroshi KikukawaStrgulc Krajšek SimonaHiroshi SatoStuart KininmonthHiroshi WakabayashiStylianos N. KounalakisHiroyuki ArakiSubhashis DasHiroyuki DateSubrata DebHiroyuki WakiguchiSuchismita RoyHisato YagiSudhir ShendeHock Siew TanSue W. LiebmanHomero Reyes De La CruzSufang LiuHong Sheng ChengSuhel GaouarHonggang ZhaoSuilan ZhengHongjun WangSujay RayHongmei ZhangSujit ShahHongxi LuoSuk Ling MaHongyan XuSumali PandeyHongyang XuSumana ChakravartyHoraţiu MoldovanSumana GhoshHortencia Silva-JímenezSumbul SaeedHosam SafaaSunbin DengHossain Ali MondalSuneet KaurHossein AziziSung Un HuhHsi Tien ChenSunil S. GangurdeHsin-Chieh LanSurbhi SharmaHsing-Hui LiSurendra RajpurohitHuafeng WangSurendra VikramHuan ZhangSuresh NarvaHuanan SuSuresh PantheeHuang LiSuresh VeeraperumalHuanjun ZhangSurya Prakash Goud PonnamHua-Tao XieSusan SanchezHuayu QiSusana Perera-ValderramaHugo MartinianoSusanne RospleszczHui CheSusanne WegmannHui JiaSusrutha Babu SukhavasiHuiching ChuangSutherland MaciverHuiling ChenSuzanna WhiteHuiming HuangSuzanne EstaphanHuizhou YangSven JelaskaHung N. MaiSvetlana KryštofováHung-Tai LeeSvetlana KurbatovaHung-Yi ChuangSvetlana V. PolevovaHunter BennettSvetozár DluholuckýHuria MarnisSwarnalatha MoparthiHyun Min JungSwati GargHyung-Bae JeonSyed NaqviIan BullSyed QadriIan G. JohnstoneSylvia García-BelenguerIbrahim M. SayedSylvie KříženeckáIbtissem TaghoutiSylvie Le BorgneIda CariatiSylwia BudzynskaIdo FeferkornSylwia OkońIftikhar AliSyoichi TashiroIgnacio BartoloméSzabolcs SzámadóIgnacio Martínez-González-MoroSzymon KaczanowskiIgnacio Ruiz-JaraboTadashi NakamuraIgor BakhmetTae Sung JungIgor KorovnikovTaekyung KimIgor OscorbinTainã Gonçalves LoureiroIgor SmojverTaiyo ToribaIgor Yu. DolmatovTakafumi ItohIhtisham BukhariTakahide HayanoIlaria CampioniTakahiko TamuraIlaria PietriniTakanobu HirosawaIldi WainerTakaoki SaneyasuIlias GatosTakashi OnoIlkka PaateroTakashi YazawaIlya EnushchenkoTakeshi SatoIlya KlabukovTakuma InagawaInayat Ur RahmanTakuma IshizakiInderjit S. SinghTakuro TobinaIndra D. SahuTalita Sarah MazzoniIndu SharmaTamal SadhukhanInés Aguinaga-OntosoTamara LazicInes AranaTamara LeskovarInês CorreiaTamás OllmannIngela MarklinderTamer AlshafieIngrid MollTamotsu HoshinoIngrid SassenhagenTanay BoseIngvar SvanbergTania RaymundoInmaculada BautistaTanja BericInnocence HarveyTanja Grubić KezeleIoanna-Katerina AggeliTanu AllenIoannis AnagnostopoulosTao HuangIoannis GiantsisTao LiIoannis GrivasTao LiuIoannis OikonomopoulosTao SuIoannis S. PaterasTara M. HenaganIonela HoteaTaranpreet KaurIonut DonoiuTareq SalehIrene CardinaliTariq MukhtarIrene DeiddaTarique HussainIrène Gallais SérézalTaro MaedaIrene Gutiérrez-CañasTasuku OkuiIrene Santos-BarriopedroTatiana BegunIrene Vázquez-DomínguezTatiana ChernovaIreneusz SowaTatiana DeniskovaIrfan A. RatherTatiana IlchibaevaIrina AtkinsonTatiana KorshunovaIrina MaslennikovaTatiana LychaginaIrineu De Brito JuniorTatiana Marisa Fernandes PatrícioIrini TsikopoulouTatiana V. KomarovaIris PlioniTatiana Y. GorpenchenkoIrma Garcia-MartinezTatjana ĆosićIsaac AmoahTatjana VujovićIsabel AbreuTatsunori IkemotoIsabel Pereira Da FonsecaTatyana R. StefanovskaIsabel Porto-HannesTawnya L. CaryIshikawa NoboruTayebeh AbediIsmael Martínez-CortésTea TomljanovićIstván EgerszegiTe-Hua HsuIstván GrigorszkyTein-Shun TsaiItzel López-RosasTeivi LaurimäeItziar Chapartegui-GonzálezTejinder PalIuga MadalinaTeleky Bernadette-EmokeIva SovadinováTeodorico De Castro RamalhoIvan A. SammutTerence LoveIvan Ayuso-FernandezTerenzio CosioIvan CundrleTeresa AdellIvan Gláucio Paulino-LimaTeresa CoutinhoIvan Gustavo Masselli Dos ReisTeresa IturriagaIvan JerkovićTeresa MougaIvan Y. IourovTeresa Rubio-TomásIvana CavelloTeru KamogashiraIvana RestovićTeruhisa KomatsuIvana ŠkrlecTeruo MiyazakiIvano ViglianteTetsuo OhyamaIveta Y. YotovaTetsuya HirataIvo HornTetsuya HoriIwona MelosikTetsuya ShiuchiIwona ŚwiątkiewiczTetsuya TaniIzabela MakalowskaTharmarajan RamprasathIzabella RządThayne KowalskiIzumi MashimaThemistoklis GiannoulisIzwan BharudinTheo SklaviadisJ. David PuettThibaut LégerJacek SiódmiakThies Henning BüscherJacek SzwedoThies Marten HeickJacobo Rodríguez-SanzThi-Huong NguyenJacopo PizzicannellaThilahgavani NagappanJacopo TalluriThilina D. SurasingheJadwiga KubicaThomas A. JeffersonJae-Hyung ParkThomas BartolomaeusJae-Kuk ShimThomas BolandJae-Young KimThomas CaspariJagdeep KaurThomas FlaggJagoda PlaczkiewiczThomas HiscockJahad SoorniThomas MarcussenJai P. RaiThomas MüllerJaime García-MenaThomas Oliver MéröJakub BiesekThomas WalugaJakub GrzesiakThomas WattsJakub GumprechtThong Ba NguyenJalal TaneeraThuareag Monteiro Trindade Dos SantosJaleel MiyanTiago DiasJam KhojastehTiago José PereiraJamal NaderiTiago RibeiroJames AllenTianhua NiuJames AndersonTianshou ZhouJames G. WilsonTianxiang GaoJames HendersonTie-Zheng WeiJames P. MuirTijana VučićJames W. McKowenTilman LamparterJan BocianowskiTimm AmendtJan DecherTimothée BaudequinJan DolfingTimothy G. HowardJan E. ConnTimothy J. JohnsJan KubesTimothy P. ClelandJan LukasTina MckayJan M. LuchtTing ZhangJan MieszkowskiTingting XiangJan PodkowińskiTino Emanuele PoloniJan SchneiderTirawat RairatJana Ambrožič-DolinšekTirtha Das BanerjeeJanaína Capelli-PeixotoToan DinhJanani ParameswaranTodd HsuJane CostaTofayel AhmedJane E. AnbinTohru SuzukiJanetta NiemannTolstenkov OlegJangPyo BaeTomás García CalvoJanko DiminićTomáš KorytářJanne SundellTomáš VyhnánekJannis FischerTomaso BottioJared MayTomasz FrączykJarosław CzyżTomasz GabryśJarosław DomaradzkiTomasz LepionkaJarosław HerbertTomasz SkawińskiJarosław J. SakTomasz StrzalaJasmina IsakovićTommaso GrecoJasmina ŽivanovićTomohiro MizobataJason BartzTomohiro YasudaJason BazilTomomi YamazakiJason C. LeppiTomoyasu FukuiJason Daniel WagganerTong GuoJason JohnstonTongqing LiJason T. C. TzenTorben StemmeJaswinder Singh MarasToshiaki ArataJavid KavousiToshiyuki SugaiJavier BotellaToyoaki OhbuchiJavier E. MercadoToyoki KozaiJavier G. OrlandiTrenton HollidayJavier Menéndez-MenéndezTrevor T. ZachariahJavier N. GelfoTriantaphyllos AkriotisJavier OlayaTristan KrapJavier Ventura-CorderoTsung-Hsien LeeJay V. PatankarTsvetelina BatsalovaJaya AseervathamTsz Tin YuJayeni Hiti-BandaralageTualar SimarmataJayesh J. AhireTuba EsatbeyogluJe Chul LeeTudor BorzaJean SavoieTuo WangJean SwingsTyler BourretJean-David MoreauTze-Huan LeiJean-Francois DesaphyTzipi KahanaJean-Marie LaplaceUfuk İlgenJean-Michel HilyUgur CakilciogluJean-Philippe PinUlises Gomez-PinedoJean-Pierre CuifUlrich SinschJeeser Alves De AlmeidaUlrike SchererJeet VariaUmar KhanJeffrey HubbardUmberto BasileJeffrey S. McFarlaneUmberto MalapelleJeffrey W. ShortUnai AzcarateJe-Keun RheeUri NirJelena IvanovićUrna KansakarJelena JovićUrszula Świderska-BurekJelena MarinkovićUrszula ZarzeckaJelena MuncanUte HabelJen-Chih TsengUttam GhoshJenna E. PruettValentin LobyshevJennifer J. KrauelValentina PaloschiJens HartungValentina PozziJeremy KiszkaValentina ŠpaničJeremy W. ChopekValeria AdamovaJérôme CrouzetValeria D′ArgenioJérôme VicenteValeria ManganelliJerry ZhuValeria MericoJerzy DzikValeria RussiniJessica MeeuwigValeria SpecchiaJessy A. SlotaValeria TrivelloneJesús D. PecoValerio GiannelliJesús Jiménez-RuizValerio IebbaJesús María López-ArrietaValério Monteiro-NetoJiabin YuValter Monteiro De Azevedo-SantosJiadong ChenVanessa MaybeckJiali YuVanessa RobitzchJian HuangVan-Giau VoJian LiVanja Čikeš-KečJian ZhouVaros PetrosyanJianbo TongVarsha ThombareJianfeng XiangVasil AtanasovJiang LiuVasileios Panagiotis E. LenisJianghong LiVasileios StathiasJianing XiVasily I. RadashevskyJianlei GuVasily KashtalapJiann-Horng LeuVassilis L. TzounakasJiaquan YuVasyl MartsenyukJiaying ZhuVed Prakash VermaJiban Kumar KunduVelimir MladenovJi-Chuan KangVenediktova NatalyaJie ChengVeniamin FishmanJie WenVenkata Reddy ChirasaniJie YangVenkatakrishnan RengarajanJie ZhaoVenkateshwari VaradharajanJiffin K. PauloseVentzislav KaramfilovJimmy Cabra-GarcíaVera KempJin SunVered NaorJingbo PangVeronica Alexandra AntipovaJingguang WeiVeronica CowlJingjing LiuVerónica Díaz-HernándezJingjing YangVeronika BókonyJingli XieVeronika Lukacs-KornekJingting YuVesa Cosmin MihaiJingxia LiuViacheslav NikolaevJingyu ZhangVibhor MishraJinhu GuoVicente MariscalJinhui WangVictor Alexandre Hardt Ferreira Dos SantosJin-Hwa JungVictor GarciaJin-Liang ZhaoVictor Hugo Gasparini NetoJinlong ZhangVictor Ivanovich SeledtsovJinn-Shing WengVíctor J. López-MadronaJinxiang XiVictor Manuel Sanchez-CorderoJiong ZhouVictor Vlad CostanJiří KvačekVictoria J. StrongJiri SkuhrovecVictoria L. MorganJiří SuchýVictoria VillegasJishnu KrishnanVida StrasserJitendra KumarVidyalakshmi SethunathJithine Jayakumar RajeswariViera KaraffovaJi-Wen XiaVika SmerduJiyang WangVikash KumarJoachim KunzVikram S. NegiJoachim NeumannVikrant RaiJoachim SiawViktor KučeraJoan VicianoViktor V. StarunovJoana CostaVilena KašubaJoana MarquezVinam PuriJoana OrtigueiraVinay Kumar IdikudaJoana V. SantosVinay Kumar TyagiJoanna KarolkiewiczVinaya Kumar KatneniJoanna KovalskiVincent NijmanJoanna KozłowskaVincent PettigroveJoão AbrantesVincent PonsJoão Guilherme De Moraes PontesVincent ReyesJoão MachadoVincenzo GrassiaJoão Paulo BritoVincenzo MarcotrigianoJoão Ricardo AraújoVincenzo MicaleJoão TrovãoVincenzo NuzziJobichen CahckoVincenzo ParrinoJochen A. MüllerVinit ShanbhagJoe K. KizhakudanVipin Chandra KaliaJoel Bueso-RódenasVirginia Martínez-RuizJoel T. KidgellVishnu RajputJohanan Christian PrasanaVišnja BesendorferJohanna Kovar-EderVitaliy KholodnyyJohanna ToivonenVitaly KozinJohannes BouchalVítězslav PlášekJohannes StraussVito Antonio Mastrochirico FilhoJohn B. VincentVito BiondiJohn BerketaVittoria RagoJohn CarusoVittoria RoncalliJohn CorkeryVivek SharmaJohn De VosVivek TanavdeJohn E. GatesyViviana MoresiJohn KomlosViviana Toro-IbacacheJohn MackrillVlad PorumbJohn ManionVladan DjordjevićJohn O. OnukwuforVladan PopovićJohn WebsterVladimir A. MyazinJohn WuVladimir DvoretskyJohnnie Van Den BergVladimir JurisicJohnny PaduloVladimir Lazarević (Sweden)Jolanta Jaroszuk-ŚcisełVladimir Lazarević (Switzerland)Jonas A. PramuditaVladimir SobolevJonas BlommeVojsava GjoniJonathan LifshitzVolker BehrendsJonathan S. MarchantVucenik IvanaJonghoe ByunVyacheslav NikulinJongmin KimW. M. Hiranya Kelum WijenayakeJordi Creus-MuncunillWaed TarrafJorge GalvezWael A. KhalilJorge Pérez-GómezWai Hang ChengJorge ToledoWai Yee LowJorge VieiraWaldemar KazimierczakJørgen KongsroWalter G. DuffyJörn TheuerkaufWalter Sánchez-SuárezJosé A. BragadaWan Mohd Rauhan Wan HussinJosé A. GonzálezWanda MączkaJosé Alberto RodriguesWann-Nian TzengJosé Antonio Cervantes-ChávezWanxue LiuJosé Antonio De Paz FernándezWaqas WakilJosé Antonio Garrido-CárdenasWasif FarooqJosé Bustos-ArriagaWasu Pathom-areeJosé Carlos Piñar-FuentesWei ChenJosé Carmelo AdsuarWei QinJosé Daniel CaminoWei SuJosé Francisco Rodríguez-VázquezWei Zhu (Chinese Academy of Sciences, Chengdu)José García Del BarrioWei Zhu (Chinese Academy of Sciences, Zhejiang)José Geraldo MillWei-Biao LiaoJosé Lino CostaWeihua DingJosé Luis Viveros-CeballosWeijia SuJosé M. Martín-DuránWei-Siang LiauJosé M. RojoWeiwei HanJosé Mariá Palacios-GarcíaWeiwei ZhangJosé Maria PratasWeiyan JiaJosé Paulo AndradeWeiyi XuJosé Rodrigo Da SilvaWen LiuJosé Trinidad Ascencio-IbáñezWen ZhangJoseph A. WestrichWen-Feng LiJoseph MarinoWenjiang DengJoseph Michael QuattroWenjun HanJoshua T. MorganWenning WangJoslyn GoberWenqiang FanJowita DrohojowskaWen-Sheng LiuJoyce E. BischoffWenzheng JiangJózef Mikołaj WiktorWeronika GonciarzJožef ŠimenkoWiebke GarrelsJuan Antonio Sánchez SáezWilawan ThongdaJuan Augusto Hernández RiveraWilliam B. MillerJuan C. Ortiz-MarquezWilliam C. MerrickJuan Diego GilbertWilliam CritchleyJuan García-Bernalt DiegoWilliam K. F. TseJuan Hernández-ÁvilaWilliam ZaragozaJuan Manuel PericasWitsanu SaisornJuan MorroneWladimir Rafael BeckJuan OchandoWładysław LasońJuan QinWłodzimierz KrzesińskiJudit KrischWojciech KiebzakJuewan KimWojciech KochJuexin WangWojciech KruszynskiJules DevauxWojciech PaslawskiJulia Sanchez-CeinosWojciech PiekoszewskiJulia Sanchez-GarridoWojciech PlazinskiJulián Cerano-ParedesWolfgang HübnerJulian Freen-van HeerenWon-Bae ParkJulian LeprinceWonchung LimJulianna KardosWonhyo SeoJulie PellerWoubit AbdelaJulien DiharceWriddhiman GhoshJulien PapaïxWuchang ZhangJulien Saint-PolWujie ZhangJulieta CarrilWuxian ShiJulieta Grajales-ConesaXavier CapóJuliette BitardXavier DallaireJulio Parra-FloresXavier MoratóJulio Plaza DíazXavier VrijdagJulio Salcedo-CastroXi LouJun LiuXiakun ChuJun PengXian-Chun ZhangJun WangXiangli ZhaoJun Yi WangXiangming TangJun YoshinagaXiangping ChuJun ZhangXiankai LuJun ZhouXiao ChenJunaidi KhotibXiao LinJuncal Gonzalez-SorianoXiao ShiJung Hoon AhnXiaohua JinJunghun ChoiXiaojun LiuJun-Ho SongXiaoke WangJunie P. WarringtonXiaokuang MaJunmei ZhangXiaoliang WangJunnan FangXiaoling LiJunping ZhangXiaolu WeiJu-Pi LiXiaomao WuJustin A. DeBlauwXiaopei GuoJustin GreenleeXiaoqian ChangJustin SchmidtXiaoshu XuJustyna BonieckaXiaoting HongJustyna Maria SokolskaXiaowen ZhangJustyna SikoraXiaoxi MengJuta KroičaXiao-Yong LiuJuvenil Enrique CaresXiaoyu DingK. G. PrasanthXiaoyu ZhangK. Gnana SheelaXichi WangK. R. AbhilashXiming GuoKai LiaoXin GaoKai LiuXin GuanKailash N. PandeyXin MuKaivalya MoluguXin-Cun WangKajohn BoonrodXingsi XueKalina DanovaXingyu WuKálmán ImreXinlei LiKalra Rajkumar SinghXinlian ZhangKalyani DhusiaXin-Ping ZhuKamal A. M. Abo-ElyousrXinxin YouKamalakar ChatlaXiongwen ChenKamil MichalikXitong ZhangKamil NelkeXiufen LiKamil RomanXiujun ZhangKampon KaeoketXiyin LiKanchana BandaraXuankun LiKangxu WangXue WangKankan WangXueying MaKaren MorenoXueying ZhangKaren MullinsXurxo Dopico CalvoKarim El-SabroutYa LiKarina JouravlevaYadong GuoKariona GrabinskaYajun XieKarl KozlowskiYaliang WangKarl VazYamini KrishnanKarlos IshacYan GuoKarol RycerzYana ZorkinaKarolina BarszczYang ChenKarolina FurtakYang ChengKaruna DixitYang LiuKata CsekőYang YangKata JuhaszYang ZhouKatarzyna BączekYangmengfan ChenKatarzyna KrawczykYanjie ZhangKatarzyna Neffe-SkocińskaYanjiong ChenKatarzyna Otulak-KoziełYan-Ming XuKatarzyna PacygaYann HeuzéKatarzyna PawlikowskaYanni SunKate EarlYannicke DauphinKateřina BělonožníkováYanwen XiongKatharine CarterYao SongKatherine ChouYao WangKatherine ElferYaseen AbbasiKatherine J. WhitehouseYasuaki NakagawaKatherine L. EllisYasuhito IshigakiKatherine LoBuglioYasuko MoriyamaKatherine SlomanYasunari MatsuzakaKathiresh ManiYaswanth KuthatiKathrin SutterYa-Tang YangKathryn Y. BurgeYavar VafaeeKatja StangeYawei LiKatja WitschasYe LiuKatrin TrentzschYen Chen TungKaustav GangopadhyayYeon-Ha KimKavindra Kumar KesariYi Chiang HsuKavita SharmaYi FengKazimierz KuliczkowskiYi SunKazufumi NakamuraYibing ZhangKazuhiro MaedaYichong FanKazuhiro P. IzawaYijian YangKazuhito ToyookaYiliang KuoKazuo KatohYiming WangKazuo KobayashiYin Ping WongKazutoshi NishijimaYin ZhaiKazuya KobayashiYinan JiangKe LiuYing LiuKeats R. ConleyYing WangKedong SongYinghui LingKeiichi FukudaYingzi ChangKeiichi KoshinakaYining JinKeith RochfortYinping LiKeizo TokuhiroYiping LuoKejd BiciYiwen MengKelley WinshipYixin HuoKelli JohnsonYixuan MaKelly S. SantangeloYoav ArnsonKelsey DawesYohan SantinKelsey MorenoYohei SawayaKelvin G. M. BrockbankYoichi EzuraKen TakasawaYoichiro HosokawaKengo SatoYoji KishiKen-ichi NonomuraYoko HasegawaKenichi YamadaYolanda Fernández-JalvoKen-ichiro MinatoYolanda García-MesaKenji HayashidaYong Sun LeeKenji IkeharaYonghe MaKennedy E. OsukaYonghong ZhangKenneth O′HalloranYonghua QinKentaro KatoYong-Ick KimKerry SmithYongjian QiuKevin D. PavelkoYongqi ShaoKevin FlahertyYongqiang WangKevin NewshamYongseok JeeKevin StevensYongsoo KimKevin WillyYongwoo JangKhadija JavedYoon-Seok ChungKhaled El-TarabilyYordan MuhovskiKhaled KhedherYoshiaki ZaizenKhalil KhamassiYoshiharu FujiiKhawar JabranYoshihiro MimakiKhurshid AhmadYoshihiro NoguchiKim Lambertsen LarsenYoshihisa HashiguchiKim M. Van VlietYoshinobu UnoKimbra KenneyYou LuKinga GawelYoung Kyun KimKinga Kostrakiewicz-GierałtYousuke TaokaKinga PlutaYu H. SunKiran LiversageYu HuangKirsten WolffYu TakeuchiKishan SambarajuYu V. BalnokinKishwar AliYuan XuKitman TsangYuanyi FengKitty H. GelbergYu-Chao WangKiyoshi TakagiYu-Chung ChiangKlára KosováYue ShengKlaus GratzYuemin BianKlaus H. HoffmannYueping HeKlaus HarzerYueying ZhouKlaus-Peter GötzYu-Guang WangKleibe De Moraes SilvaYuichi TsuchiyaKoichi FujisawaYuji HirowatariKoji TsuchidaYuji MatsumotoKok Zhi LeeYuji MoritaKoki MakabeYuki FukudaKondalarao BankapalliYukiko MatsudaKondareddy CherukulaYulei WeiKongju ZhuYung-Chieh ChangKongpan LiYun-Wen ZhengKonrad FiedlerYupin PhasukKonstantin ChekanovYuri TokarevKonstantin N. YaryginYusi CuiKonstantinos A. GatzoulisYusuf OladosuKonstantinos FeidantsisYusuf SertKonstantinos GkagkavouzisYu-Wei FangKonstantinos NatsisYuxiang LiKonstantinos TsagarakisYves DenizotKosmas NathanailidesYves HenryKostas KougioumoutzisZacharias PapadakisKosuke KajiZachary S. ClaytonKosuke YoshidaZain-ul AbadinKouichi KawamuraZakaria Hossain ProdhanKoushik BrahmachariZarin MachandaKrekwit ShinlapawittayatornZarrin BasharatKrishna KarmakarZdeňka BendováKrishnamoorthy SrikanthZehuan LiaoKrishnan M. DhandapaniŽeljko D. SavkovićKrishnan RathinasamyZeno GuardiniKristine M. KrajnakZhan ShuKristof DeseureZhanqi ZhaoKristyn K. VoegeleZhaoming QiKrithika RaoZhe ZhangKrystyna Pierzchała-KoziecZhen ChenKrzysztof ChmielowiecZhen DaiKrzysztof Durkalec-MichalskiZhen ShuKrzysztof FlisikowskiZhenbing WuKrzysztof FrączekZheng WangKrzysztof KrawczykZhengfei WangKrzysztof KudZhenya IlievaKrzysztof PolowyZhicheng CuiKrzysztof R. KarszniaZhidong ZhouKrzysztof TomczykZhiguo ZhangKuang-Tai KuoZhijian JiangKuan-Hsuan ChenZhili PangKuldeep AttriZhipeng CaoKuldeep GuptaZhiqiang WuKun HuangZhiqiu XiaKundan SenguptaZhishuai HouKunlin WeiZhiwen DingKuntheavy Ing-LorenziniZhiying LuKuo-Shyan LinZhiyong LiuKurt PfannkucheZhongfei HanKusum DhakarZhongjian ChengKwanuk LeeZhongling ShangKyle BurghardtZhongqi MiaoKyojiro N. IkedaZhongzhi ChenKyuBum KwackZhuan LiKyung In BaekZhu-Liang YangLadislav ČepelkaZiad Al-DwairiLaia Just-BorràsZin Z. KhaingLamine Baba-MoussaZixiao GuoLan XiongZlatko PuškadijaLana ZorićZlatozar BoevLanre MorenikejiZoltán BátoriLapo TurriniZonghang ZhangLara CostantiniZongjie WangLara K. AbramowitzZoya MarinovaLarisa LitvinovaZsuzsanna FarkasLarry J. GrabauZsuzsanna LengyelLars MalmströmZunlong KeLászló HéjaZunyao WangLaudí Cunha LeiteZurisaday Santos-JimenezLaura Aracely Contreras-AnguloZuzana AndrejčákováLaura BowdenZuzana HeřmanováLaura Gheucă SolovăstruZuzana Kronekova


